# Cytomegalovirus anterior uveitis in immunocompetent individuals following topical prostaglandin analogues

**DOI:** 10.1186/1869-5760-3-55

**Published:** 2013-07-09

**Authors:** Kalpana Babu, Gowri Jaydev Murthy

**Affiliations:** 1 Vittala International Institute of Ophthalmology, Bangalore, India; 2 Prabha Eye Clinic and Research Centre, 504, 40th Cross, Jayanagar 8th Block, Bangalore 560070, India

**Keywords:** Cytomegalovirus, Anterior uveitis, Prostaglandin analogues, Glaucoma

## Abstract

**Background:**

The aim of this study is to report two interesting cases of cytomegalovirus (CMV) anterior uveitis following topical prostaglandin analogue administration for glaucoma. Two retrospective case studies are presented.

**Findings:**

A 40-year-old immunocompetent lady with a history of Fuchs heterochromic iridocyclitis with secondary glaucoma in the right eye since 2005 was diagnosed to have CMV anterior uveitis by a multiplex polymerase chain reaction (PCR) in 2009. She developed a reactivation of anterior uveitis following the addition of latanoprost 0.005% eye drops unknowingly by her local ophthalmologist. The pattern of endothelial deposits seen with this reactivation of uveitis was different from that seen in earlier or in subsequent reactivations. A 46-year-old immunocompetent lady with a history of primary open-angle glaucoma and no history of uveitis presented with anterior uveitis with medium-sized keratic precipitates following administration of travatoprost 0.004% eye drops. In both cases, the CMV antigen was demonstrated in the aqueous by multiplex PCR at the time of reactivation. Both cases required treatment with dexamethasone eye drops, ganciclovir 1% gel and oral valganciclovir for the control of inflammation along with antiglaucoma medications.

**Conclusions:**

We report two immunocompetent cases with the development of CMV-related anterior uveitis following administration with prostaglandin analogues. These cases increase the awareness of CMV anterior uveitis in immunocompetent individuals and the need to use prostaglandin analogues with caution.

## Findings

### Introduction

Topical prostaglandin analogues used to treat glaucoma are known to cause exacerbations of uveitis [1,2]. Reactivation of herpex simplex keratitis following its use has been described in the literature [3]. We report two interesting cases of cytomegalovirus (CMV) anterior uveitis in immunocompetent individuals following administration of topical prostaglandin analogues. To our knowledge, this is the first case series of reactivation of CMV following topical prostaglandin analogues (Medline search).

#### Case 1

**Figure 1:**
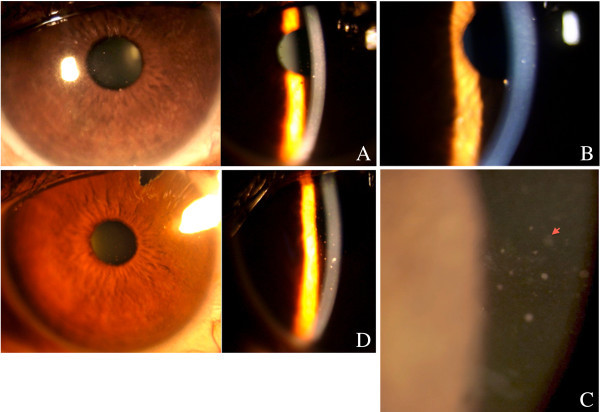
**Slit-lamp examination of the right eye of case 1.** Showing **(A)** iris moth-eaten pattern with keratic precipitates located centrally, **(B)** pigmented keratic precipitate with a clear halo located centrally in a quiet eye, **(C)** multiple map-like lesions (arrow) and fine and medium-sized keratic precipitates on the endothelium following latanoprost 0.005% eye drops and **(D)** reactivation of anterior uveitis with fine to medium-sized keratic precipitates post-filtering surgery.

A 40-year-old immunocompetent lady initially diagnosed to have Fuchs heterochromic iridocyclitis with secondary glaucoma (OD) in 2005 (Figure 1A) had a history of ocular inflammations with fluctuations in intraocular pressure (IOP) for which she was using fluorometholone eye drops (on and off) and antiglaucoma medications (betaxolol 0.5% twice a day). In 2009, an aqueous tap done for persistently high IOP (42 mmHg) showed positive for CMV antigen by a multiplex PCR and negative for herpes simplex virus (HSV), varicella zoster virus (VZV), rubella, chikungunya, toxoplasma and *Mycobacterium tuberculosis* (MTb). Serology for CMV IgG was positive but negative for CMV IgM. A moth-eaten appearance of the iris with no posterior synechiae, 1 + AC reaction (SUN) and medium-sized pigmented keratic precipitates located centrally with a surrounding halo was seen on slit-lamp examination. The inflammation and IOP stabilized with oral valganciclovir (900 mg bid for 3 weeks followed by 450 mg bid for 1 month), dexamethasone eye drops (tapered weekly from an initial dose of six times a day) and ganciclovir eye gel 0.15% five times a day. Since then, she has been on a fixed combination of 0.2% brimonidine and 0.5% timolol eye drops twice a day. In 2010, she was shifted to latanoprost 0.005% eye drops due to a skin allergy to the above. A quiet eye with a pigmented central keratic precipitate with a clear halo and no AC reaction (Figure 1B) was recorded before starting the drops. She came back with blurring of vision (OD) 4 days later. Her best-corrected visual acuity (BCVA) was 6/6 (OU). Slit-lamp examination (OD) showed multiple map lesions on the endothelium along with fresh, fine and medium-sized keratic precipitates (Figure 1C) and an IOP of 50 mmHg. Aqueous tap (OD) was again positive for CMV antigen by multiplex PCR. Her uveitis and IOP stabilized with discontinuation of the drug and administration of dexamethasone eye drops. She eventually required a filtering surgery. She developed a reactivation once after the filtering surgery (Figure 1D), which settled with oral valganiciclovir, dexamethasone eye drops and ganciclovir gel. At 2 years follow-up, ocular inflammation and IOP are stable.

#### Case 2

**Figure 2:**
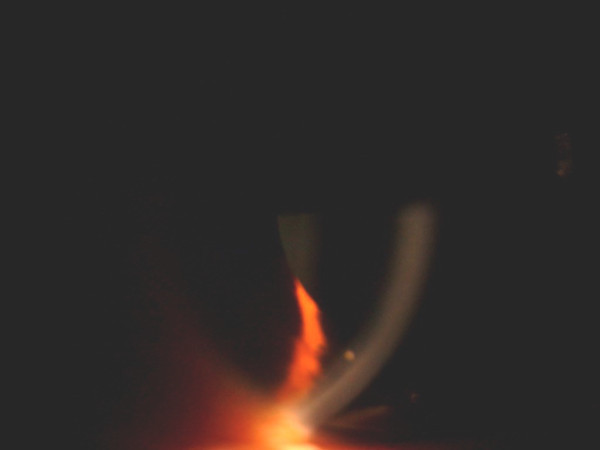
**Slit-lamp examination of the right eye of case 2.** Showing inferiorly located medium-sized keratic precipitate after travoprost 0.004% eye drops.

A 46-year-old immunocompetent lady with a history of primary open-angle glaucoma (OU) since 2006 presented with blurring of vision (OD) after starting travoprost 0.004% eye drops for the right eye a week ago. She had no history of uveitis. At the time of presentation, her BCVA was 6/6 (OU). Slit-lamp evaluation (OD) showed medium-sized keratic precipitates located inferiorly and centrally (Figure 2) and an IOP of 52 mmHg. Multiplex PCR on the aqueous tap (OD) showed a positive CMV antigen and negative for HSV, VZV, rubella, chikungunya, toxoplasma and MTb. Serology for CMV IgG was positive but negative for CMV IgM. Her uveitis and IOP settled with discontinuation of travoprost 0.004%, addition of dexamethasone eye drops and oral valganciclovir (900 mg bid for 3 weeks followed by 450 mg bid for 1 month). At 6 months follow-up, her inflammation and IOP are stable.

## Discussion

CMV is known to cause anterior uveitis in immunocompetent patients [4-6]. Reactivation of CMV anterior uveitis has been reported following fluocinolone-sustained steroid drug delivery implant [4]. Our first case had a prior history of CMV anterior uveitis and was started on latanoprost leading to reactivation of anterior uveitis. The pattern of multiple map-like lesions on the endothelium along with keratic precipitates was seen only during the reactivation following the prostaglandin analogues (Figure 1C was different compared to that seen in Figure 1A,D). The second case did not have a history of uveitis and developed a CMV anterior uveitis only after administration of travoprost eye drops. In both cases, the serology showed increased titres of CMV IgG. We speculate that CMV remains latent in the anterior chamber in some individuals, and this may reactivate when the local milieu is altered, such as with the administration of prostaglandin analogues. Ophthalmologists must be aware of this presentation of CMV anterior uveitis following usage of prostaglandin analogues.

## Competing interests

The authors declare that they have no competing interests.

## Authors’ contributions

KB and GJM carried out the diagnosis and management of these cases and drafted the manuscript. Both authors read and approved the final manuscript.
